# Impact of Sema3A Interference on Cerebellum-Dependent Motor Associative Learning and Memory

**DOI:** 10.3390/ijms27125304

**Published:** 2026-06-11

**Authors:** Geoffrey-Alexander Gimenez, Sarah Van Der Zwaag, Cynthia M. Geelen, Melissa Van Hemert, Jop Vreeken, Fred de Winter, Cathrin B. Canto, Daniela Carulli, Chris I. De Zeeuw, Joost Verhaagen

**Affiliations:** 1 Department of Neuroregeneration, Netherlands Institute for Neuroscience, Royal Netherlands Academy of Arts and Sciences, Meibergdreef 47, 1105 BA Amsterdam, The Netherlands; g.gimenez@nin.knaw.nl (G.-A.G.); f.de.winter@nin.knaw.nl (F.d.W.); d.carulli@nin.knaw.nl (D.C.); 2 Department of Cerebellar Coordination & Cognition, Netherlands Institute for Neuroscience, Royal Netherlands Academy of Arts and Sciences, Meibergdreef 47, 1105 BA Amsterdam, The Netherlands; sarahvdzwaag@hotmail.com (S.V.D.Z.); c.geelen@nin.knaw.nl (C.M.G.); m.s.hemert@gmail.com (M.V.H.); j.vreeken@amsterdamumc.nl (J.V.); c.canto@nin.knaw.nl (C.B.C.); c.de.zeeuw@nin.knaw.nl (C.I.D.Z.); 3 Department of Neuroscience, Erasmus MC, 3015 GD Rotterdam, The Netherlands; 4 Center for Neurogenomics and Cognitive Research, Amsterdam Neuroscience, Vrije Universiteit Amsterdam, 1081 HV Amsterdam, The Netherlands

**Keywords:** semaphorin 3A, perineuronal nets, eyeblink conditioning, cerebellar nuclei, neuroplasticity

## Abstract

Semaphorin 3A (Sema3A), a known axon chemorepulsive protein during development, is localised in perineuronal nets (PNNs) in the adult brain. PNNs are condensed aggregates of extracellular matrix molecules surrounding specific types of neurons, which regulate neuroplasticity and memory. However, the role of PNN-associated Sema3A in these processes remains unclear. To address this topic, we investigated the contribution of Sema3A to cerebellum-dependent learning and memory in adult mice using the eyeblink conditioning (EBC) paradigm. We interfered with Sema3A signalling by employing: (i) a molecular approach, in which secreted Sema3A receptors (neuropilin-1 bodies) were expressed in the anterior interposed nuclei (AIN) via viral vector injection; and (ii) a genetic approach, using mutant mice with impaired Sema3A signalling (K108N mice). Mice expressing neuropilin-1 bodies showed reduced EBC performance at the beginning of the memory retention phase. However, increased inflammation was found in the AIN of these mice, challenging the interpretation of these findings. K108N mice showed enhanced EBC performance at the beginning of the memory retention phase. No synaptic structural changes were detected in the AIN of K108N mice at the end of the EBC paradigm. Based on our findings in K108N mice, constitutively altered Sema3A signalling is associated with subtle improvement in cerebellar memory.

## 1. Introduction

Semaphorin 3A (Sema3A) is a chemorepulsive protein involved in axon guidance during neural development [1,2,3,4]. Sema3A exerts its effect on target cells via a transmembrane receptor complex composed of neuropilin-1 (NRP1) and plexinA1/4 [5,6,7,8,9]. In the adult central nervous system, *Sema3A* mRNA is expressed in neuronal subsets across brain regions [10], whereas Sema3A protein is primarily localised in perineuronal nets (PNNs) [11]. PNNs are dense assemblies of extracellular matrix (ECM) molecules that enwrap the soma, proximal dendrites and axon initial segment of certain classes of neurons [12,13,14]. Sema3A binds to specific chondroitin sulphate motifs of the chondroitin sulphate proteoglycans (CSPGs), which are major ECM components of the PNN [15,16,17,18]. PNNs form in early postnatal life during critical periods, developmental time windows characterised by heightened levels of neuroplasticity [19,20,21,22]. PNNs restrict plasticity at the end of critical periods [19,23,24]. PNNs control the electrophysiological properties of PNN-enwrapped neurons as well as receptor availability on the post-synaptic membrane [25,26,27]. In addition, PNNs contribute to protecting neurons they surround from oxidative stress and influence local ion homeostasis [28,29,30,31,32]. At the network level, PNNs have been suggested to be involved in short- and long-term memory consolidation [23,33,34,35]. For a detailed overview of PNN function, see reviews [14,36,37,38].

In the cerebellum, PNNs are present around Golgi neurons in the cerebellar cortex and around glutamatergic neurons in the cerebellar nuclei (CN) [39]. A more diffuse semi-organised PNN-like pattern is present around granule cells, Purkinje cells and molecular layer interneurons [39,40]. The majority of CN PNNs are Sema3A positive [41]. In plasticity-associated conditions, such as following enriched environmental stimulation, the expression of PNNs [42] and PNN-associated Sema3A [41] around CN neurons is decreased. Consistent with this, a decrease in PNNs has also been observed in the CN of mice after delay eyeblink conditioning (EBC) [43]. Delay EBC is a cerebellum-dependent form of Pavlovian associative motor learning in which a neutral conditioned stimulus (CS), such as an LED light, is paired with an aversive unconditioned stimulus (US), such as an air puff, at a fixed interval, with both stimuli having specific durations and co-terminating. Repeated presentations of the paired stimuli lead to the emergence of a well-timed eyeblink conditioned response (CR) [44,45,46]. The cerebellar nucleus primarily involved in EBC is the anterior interposed nucleus (AIN). This has been consistently demonstrated across species using multiple approaches—lesioning, cooling and lidocaine or muscimol infusion—which all impair the AIN and consequently disrupt CRs [44,45]. Digestion of PNN chondroitin sulphates in the AIN, using chondroitinase ABC, improves EBC learning by increasing the percentage of conditioned responses (%CR) [43,47] and the fraction of eyelid closures (FEC) [43]. Although it has been demonstrated that PNNs control cerebellum-dependent learning and memory, the contribution of PNN-associated Sema3A to EBC remains unknown.

In this article we investigated the role of PNN-associated Sema3A in EBC learning and memory. We interfered with Sema3A signalling using two different approaches. First, a local molecular approach, injecting an adeno-associated viral vector (AAV) in the AIN expressing a secreted form of the transmembrane NRP1 receptor, referred to as NRP1 receptor bodies. This modified NRP1 receptor contains a mutation (Y297A) that significantly reduces binding to the vascular endothelial growth factor, another NRP1 ligand [48], and is fused to a human IgG Fc fragment, replacing the transmembrane domain, to promote dimerization [49]. These secreted NRP1 receptor bodies act as scavengers of Sema3A and interfere with the signalling that normally occurs via the endogenous transmembrane NRP1 receptor [49]. Second, a global genetic approach, exploiting a mouse line with a point mutation (K108N) in the *Sema3A* gene (Sema3A^K108N/K108N^ mice, named K108N mice in this article), which alters the interaction between Sema3A and its receptor complex and strongly decreases its repulsive activity [50]. Motor associative learning, memory consolidation and relearning were assessed using EBC in mice expressing NRP1 receptor bodies in the AIN and in K108N mice. Following completion of the EBC protocol, changes in the number and size of synaptic terminals in the AIN were investigated in both the local molecular approach and the global genetic approach to determine whether Sema3A interference affected structural plasticity.

## 2. Results

### 2.1. Expression of NRP1 Receptor Bodies in the AIN Decreases EBC Performance at the Beginning of the Memory Retention Phase

To interfere with Sema3A signalling, we bilaterally injected into the AIN of adult wild-type (WT) C57BL/6J mice an AAV8-CMV-NRP1(Y297A)-Fc-WPRE (AAV-NRP1) viral vector to express NRP1 receptor bodies (NRP1 mice). Control WT mice were injected with an AAV8-CMV-Fc-WPRE (AAV-Fc) viral vector (Fc mice). After 4 weeks, these mice underwent an EBC protocol to assess their motor associative learning and memory. Non-injected WT mice were used as further controls for EBC performance to exclude potential interference of the viral vector injection (WT mice). The EBC protocol consisted of three distinct phases: learning, memory retention, and relearning. During the learning and relearning phases, mice were trained with paired trials (CS and US combined) to learn/relearn the association of the US with the CS. CS trials were given every 10 paired trials to evaluate the progression of learning/relearning. During the memory retention phase, only CS trials were given to measure the consolidation of the associated motor learning. The EBC performance of mice was evaluated by measuring their %CR, FEC, CR onset time (CRonset time) and CR maximum amplitude time (CRmaxA time) per session for CS and paired trials.

**Figure 1:**
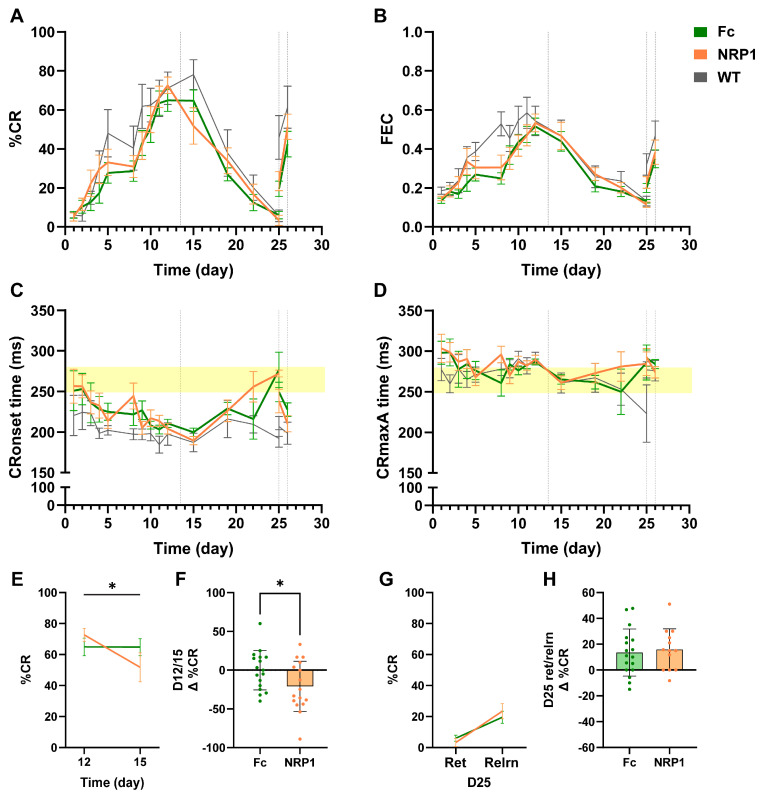
Expression of NRP1 receptor bodies in the AIN decreases EBC performance at the beginning of the memory retention phase. (**A**–**D**) EBC performance of Fc, NRP1 and WT mice, shown as (**A**) %CR, (**B**) FEC, (**C**) CRonset time and (**D**) CRmaxA time for CS trials. Dashed lines separate learning (D1–12), memory retention (D15–25) and relearning (D25–26) phases (learning phase: Fc *N mice* = 17, NRP1 *N mice* = 17, WT *N mice* = 7; memory retention phase: Fc *N mice* = 17, NRP1 *N mice* = 16, WT *N mice* = 7; relearning phase: Fc *N mice* = 17, NRP1 *N mice* = 15, WT *N mice* = 7). Yellow block indicates the US period (**C**,**D**). (**E**,**F**) %CR (**E**; Fc *N mice* = 17, NRP1 *N mice* = 17) and Δ %CR (**F**; Fc *N mice* = 17, NRP1 *N mice* = 16) for CS trials during the transition from learning to memory retention (D12/15) in Fc and NRP1 mice. (**G**,**H**) %CR (**G**; Fc *N mice* = 17, NRP1 *N mice* = 15) and Δ %CR (**H**; Fc *N mice* = 17, NRP1 *N mice* = 13) for CS trials during the transition from memory retention to relearning (D25 ret/relrn) in Fc and NRP1 mice. (**A**–**E**,**G**) Data are shown as mean ± SEM. (**F**,**H**) Data are shown as mean ± SD. * *p* < 0.05.

During the learning phase (day 1–12), no significant differences were found for CS trials among Fc, NRP1 and WT mice in the %CR, FEC, CRonset time and CRmaxA time (Figure 1A–D). However, analysis of paired trials revealed significant differences in the %CR and the CRonset time among the three groups, along with a trend for the CRmaxA time (time x condition, %CR: *p* = 0.023, F (18, 338) = 1.81, CRonset time: *p* = 0.002, F (18, 314) = 2.34, condition, CRmaxA time: *p* = 0.069, F (2, 38) = 2.87; Supplementary Figure S1A–D). Pairwise comparison for paired trials within individual days revealed a significantly faster CRonset time on day (D) 1 for Fc mice when compared to WT mice (*p* = 0.042, 95% CI = [−93.92, −0.97]; Supplementary Figure S1C). The FEC for paired trials remained non-significantly different across conditions (Supplementary Figure S1B). During the memory retention phase (day 15–25), the %CR, FEC, CRonset time and CRmaxA time for CS trials did not significantly differ between Fc, NRP1 and WT mice (Figure 1A–D). However, there was a trend between conditions for the CRonset time (time x condition, *p* = 0.069, F (6, 61) = 2.08, condition, *p* = 0.081, F (2, 37) = 2.69; Figure 1C). Pairwise comparison for CS trials within individual days showed a trend towards a slower CRonset time for Fc mice when compared to WT mice on D25 (*p* = 0.081, 95% CI = [−7.85, 176.8], Figure 1C). During the relearning phase (day 25 and 26), no significant differences were found among the three groups in the FEC, the CRonset time and the CRmaxA time for CS trials (Figure 1B–D). However, a trend was observed in the %CR when comparing Fc, NRP1 and WT mice (condition, *p* = 0.069, F (2, 36) = 2.88; Figure 1A). Pairwise comparison for CS trials on individual days revealed a trend towards a lower %CR for Fc mice when compared to WT mice on D25 (*p* = 0.088, 95% CI = [−54.97, 2.31], Figure 1A). Analysis of paired trials revealed significant differences in the %CR across groups, along with a trend in FEC (condition, %CR: *p* = 0.039, F (2, 36) = 3.55, FEC: *p* = 0.092, F (2, 36) = 2.55; Supplementary Figure S1A,B). However, pairwise comparison for paired trials on individual days showed a trend towards a lower %CR for Fc mice when compared to WT mice on D26 (*p* = 0.097, 95% CI = [−52.56, 2.64]; Supplementary Figure S1A). The CRonset time and the CRmaxA time for paired trials remained non-significantly different across conditions (Supplementary Figure S1C,D).

Given the absence of clear differences between Fc and NRP1 mice within each experimental phase, we investigated the %CR of CS trials across transition phases for Fc and NRP1 mice: from learning to memory retention (D12/15) and from memory retention to relearning (D25 ret/relrn). The first transition (D12/15) showed significantly different %CR between Fc and NRP1 mice. Fc mice maintained stable %CR (~64%), while NRP1 mice exhibited a decrease in %CR from ~64% to ~51% (time x condition, *p* = 0.047, F (1, 31) = 4.3; Figure 1E). However, no changes for pairwise comparison on individual days were found for the D12/15 transition (Figure 1E). To confirm this result, we measured the performance Δ of %CR (Δ %CR) for each mouse between D12 and D15. NRP1 mice performed significantly worse than Fc mice over the D12/15 transition (~−21%; *p* = 0.048, t = 2.06, df = 31; Figure 1F). The D25 ret/relrn transition did not reveal significant differences between Fc and NRP1 mice for the %CR or Δ %CR (Figure 1G,H).

Overall, these results indicate that expression of NRP1 receptor bodies in the AIN affects the beginning of the memory retention phase, suggesting that Sema3A may subtly promote the consolidation or expression of the conditioned eyeblink memory.

### 2.2. Expression of NRP1 Receptor Bodies Decreases the Density of Gephyrin Puncta Along the Somatic Membrane of AIN Neurons After the Relearning Phase

**Figure 2:**
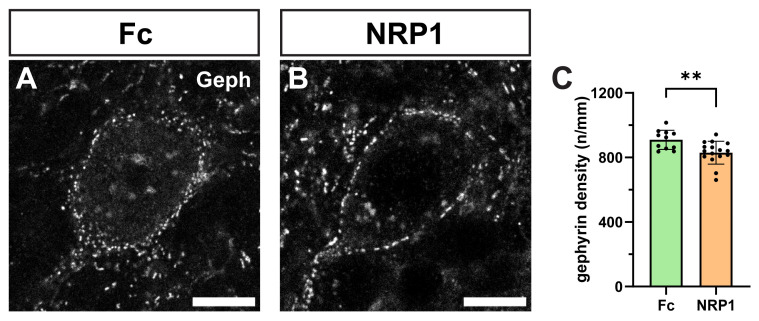
Expression of NRP1 receptor bodies decreases the density of gephyrin puncta along the somatic membrane of AIN neurons after the relearning phase. (**A**,**B**) Example of gephyrin staining in an AIN neuron of Fc (**A**) and NRP1 (**B**) mice, located in the area of highest transduction. (**C**) Gephyrin density in Fc and NRP1 mice (Fc *N mice* = 11, NRP1 *N mice* = 16). Scale bar: (**A**,**B**): 10 µm. Data are shown as mean ± SD. ** *p* < 0.01.

To understand the physiological mechanisms underlying the subtle effect of Sema3A interference on EBC using NRP1 receptor bodies, we examined whether structural changes occur in the AIN of NRP1 mice when compared to Fc mice after EBC relearning. A previous study demonstrated that growth cones of dorsal root ganglion neurons failed to collapse when exposed to a combination of Sema3A and NRP1 receptor bodies, whereas they collapsed in the presence of Sema3A alone [49]. Additionally, a study on synaptic modulation in WT mice following EBC reported increased gephyrin levels in the AIN of conditioned mice when compared to pseudo-conditioned mice [51]. Gephyrin is a widely used marker for inhibitory synapses, as it is essential for clustering glycine and GABA_A_ receptors at the post-synaptic sites [52]. Thus, we quantified the density of gephyrin^+^ puncta along the somatic membrane of AIN neurons. Gephyrin density was significantly reduced in NRP1 mice when compared to Fc mice (*p* = 0.005, t = 3.04, df = 25; Figure 2A–C). Previous work on EBC reported structural changes in the AIN in the ipsi- and contralateral side of the US [53]. Thus, we quantified gephyrin in the left and right AIN separately and found that gephyrin density was significantly reduced in both hemispheres of NRP1 mice when compared to Fc mice (left AIN: *p* = 0.004, t = 3.18, df = 22, right AIN: *p* = 0.012, t = 2.74, df = 22; Supplementary Figure S2A,B).

### 2.3. Expression of NRP1 Receptor Bodies in the AIN Leads to Inflammation

**Figure 3:**
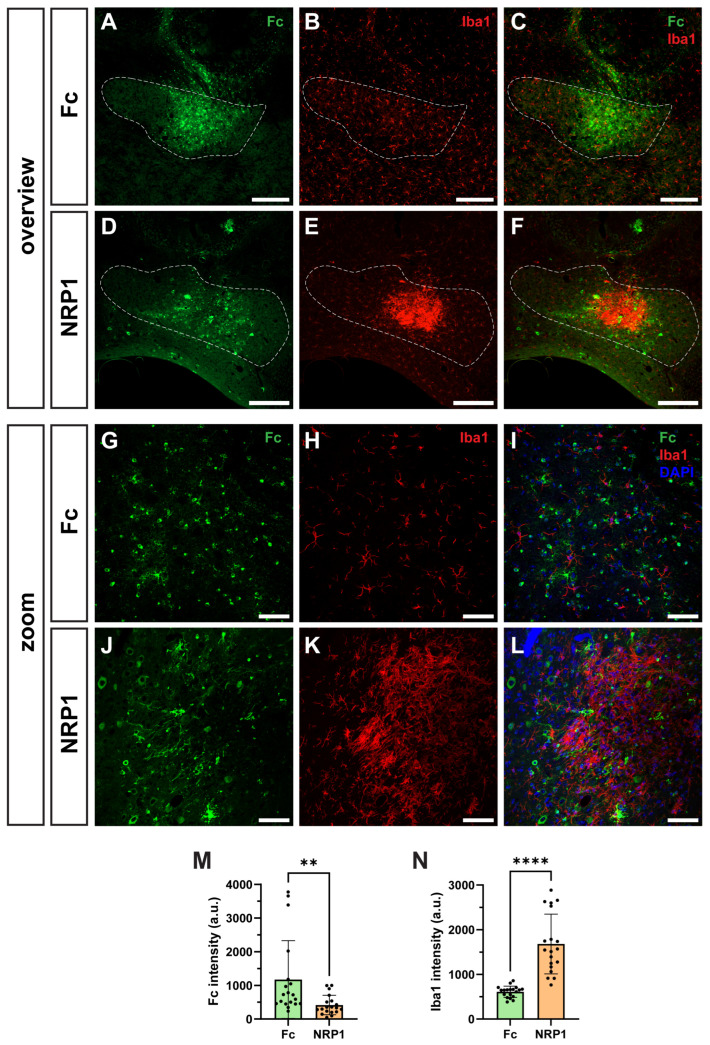
Expression of NRP1 receptor bodies in the AIN leads to inflammation. (**A**) Example images of Fc staining in the AIN of Fc mice. (**B**,**C**) Iba1 staining of Fc mice revealed normal distribution of microglia-like cells in the area of highest transduction and its periphery. (**D**) Example image of Fc staining in the AIN of NRP1 mice. (**E**,**F**) Iba1 staining of NRP1 mice revealed inflammation, with particularly abundant staining in the area of highest transduction. (**G**–**I**) Zoomed-in images of panels (**A**–**C**) (including DAPI), respectively. (**J**–**L**) Zoomed-in images of panels (**D**–**F**) (including DAPI), respectively. (**M**) Quantification of Fc intensity in Fc and NRP1 mice. (**N**) Quantification of Iba1 intensity in Fc and NRP1 mice (Fc *N mice* = 19, NRP1 *N mice* = 19). AIN outlined by dashed line in (**A**–**F**). Scale bar: 250 µm (**A**–**F**); 60 µm (**G**–**L**). Data are shown as mean ± SD. ** *p* < 0.01, **** *p* < 0.0001.

Prior to performing a staining for gephyrin, a test staining was performed on some Fc and NRP1 mice using a mouse anti-gephyrin primary antibody, followed by a fluorophore-conjugated, non-subtype-specific anti-mouse IgG secondary antibody. Alongside gephyrin^+^ puncta, gephyrin^+^ microglia-like cells were observed only in NRP1 mice (Supplementary Figure S3A). This microglia-like pattern disappeared when a subtype-specific anti-mouse IgG1 secondary antibody was used (Supplementary Figure S3B), suggesting that endogenous IgGs, naturally produced and secreted by B lymphocytes, were bound to microglia Fcγ receptors via their Fc region and were subsequently recognised by the non-subtype-specific secondary antibody. This nonspecific signal in NRP1 tissue suggested an increase in microglia Fcγ receptors, increasing the likelihood of nonspecific secondary antibody binding. Therefore, we stained Fc and NRP1 mice for Iba1 to assess whether NRP1 receptor bodies triggered the immune system in the AIN by means of microglia and macrophage reactivity. Additionally, we performed Fc staining to correlate immune cell reactivity with Fc expression levels. Both Fc and NRP1 mice showed strong transduction in the AIN with AAV-Fc (Figure 3A) or AAV-NRP1 (Figure 3D) vectors. An Fc^+^ signal was found in neurons and in the ECM in both Fc and NRP1 mice (Figure 3G,J). Fc expression levels were significantly higher in the AIN of Fc mice compared with NRP1 mice when both sides were analysed together (*p* = 0.001, U = 72; Figure 3M). Separate analysis of the left and right AIN confirmed transduction on both sides in Fc and NRP1 mice (Supplementary Figure S4A,C). Accordingly, Fc expression levels were significantly higher in Fc mice than in NRP1 mice on either side (left AIN: *p* = 0.036, U = 71, right AIN: *p* = 0.0002, U = 37; Supplementary Figure S4A,C). This difference in transgene expression levels was expected since the size of the CMV-Fc-WPRE sequence is smaller than that of the CMV-NRP1(Y297A)-Fc-WPRE (2950 bp vs. 5538 bp, respectively), leading to more frequent transcription of the transgene in transduced cells. As suggested by the nonspecific secondary antibody binding to microglia-like cells in NRP1 mice described above, Iba1^+^ cells were more abundant in the AIN of NRP1 mice when compared to Fc mice (Figure 3B,E), particularly in areas of highest transduction (Figure 3C,F). The morphology of Iba1^+^ cells in NRP1 mice suggested that the cells were microglia in an inflammatory reactive state (Figure 3K) and were highly condensed in the transduction area (Figure 3L). In contrast, Iba1^+^ cells in Fc mice appeared to be in a resting state (Figure 3H) and were homogeneously scattered across the AIN (Figure 3B,I). Quantification of Iba1 immunostaining showed significantly higher intensity in NRP1 mice when compared to Fc mice (*p* < 0.0001, t = 6.86, df = 19.26; Figure 3N). A similar outcome was observed when separating the left and right AIN of Fc and NRP1 mice (left AIN: *p* < 0.0001, t = 5.92, df = 14.83, right AIN: *p* = 0.0002, t = 4.97, df = 15.04; Supplementary Figure S4B,D). Altogether, the inflammation found in NRP1 mice may confound the interpretation of the behavioural and histological findings described above.

### 2.4. Genetically Interfering with Sema3A Signalling Increases EBC Performance at the Beginning of the Memory Retention Phase Following the Learning Phase

After investigating the effects of molecular interference of Sema3A signalling on cerebellum-dependent motor associative learning and memory, we examined the impact of genetically interfering with Sema3A signalling. Specifically, we employed a genetic mouse model carrying a point mutation in the *Sema3A* gene (K108N mice), which disrupts the interaction between Sema3A and its receptor complex, thereby reducing its repulsive activity [50]. Homozygous K108N mice and wild-type C57Bl/6J control mice (WT mice) underwent an EBC protocol similar to the one described above, with the addition of an extra memory retention phase (memory retention-2 phase: D29), introduced after the relearning phase. As in the memory retention-1 phase, only CS trials were given during the memory retention-2 phase. This extra phase, consisting of one day, was added to correlate the histology with the memory retention phase, as EBC on NRP1 mice suggested an effect of Sema3A at the beginning of the memory retention phase.

**Figure 4:**
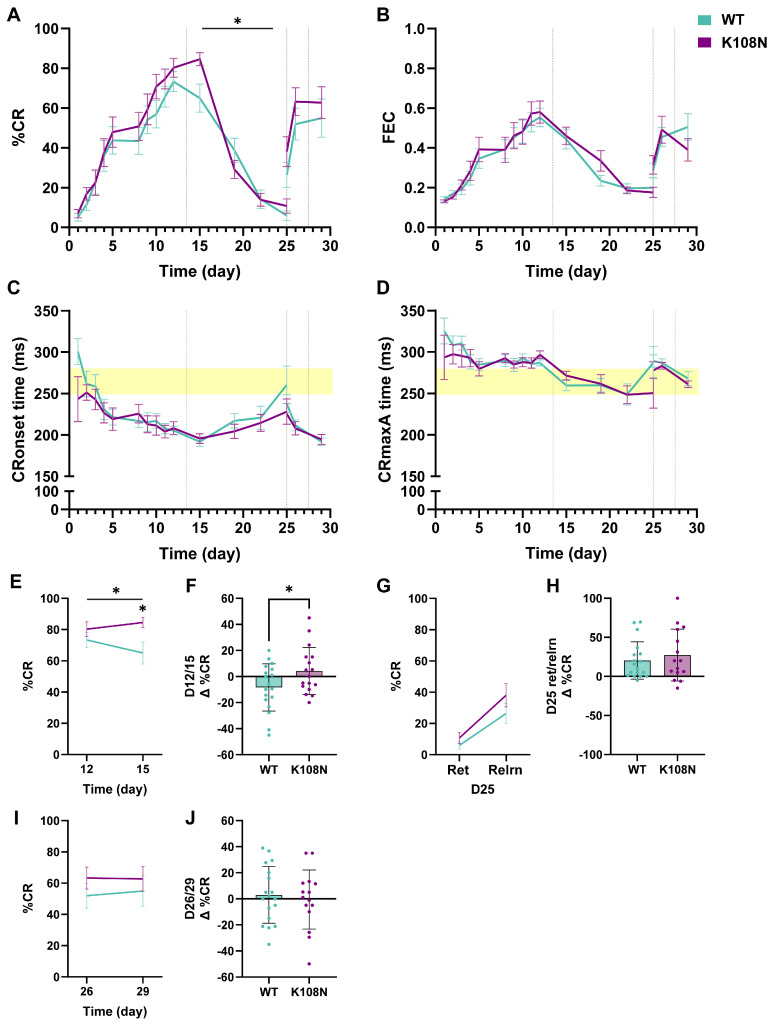
Genetically interfering with Sema3A signalling increases EBC performance at the beginning of the memory retention phase following the learning phase. (**A**–**D**) EBC performance of WT and K108N mice, shown as (**A**) %CR, (**B**) FEC, (**C**) CRonset time and (**D**) CRmaxA time for CS trials. Dashed lines separate learning (D1–12), memory retention (D15–25), relearning (D25–26) and memory retention-2 (D29) phases (learning phase: WT *N mice* = 18, K108N *N mice* = 17; memory retention-1 phase: WT *N mice* = 18, K108N *N mice* = 17; relearning phase: WT *N mice* = 18, K108N *N mice* = 15; memory retention-2 phase: WT *N mice* = 18, K108N *N mice* = 15). Yellow block indicates the US period (**C**,**D**). (**E**,**F**) %CR (**E**) and Δ %CR (**F**) for CS trials during the transition from learning to memory retention-1 (D12/15) in WT and K108N mice (WT *N mice* = 18, K108N *N mice* = 17). (**G**,**H**) %CR (**G**) and Δ %CR (**H**) for CS trials during the transition from memory retention-1 to relearning (D25 ret/relrn) in WT and K108N mice (WT *N mice* = 18, K108N *N mice* = 15). (**I**,**J**) %CR (**I**) and Δ %CR (**J**) for CS trials during the transition from relearning to memory retention-2 (D26/29) in WT and K108N mice (WT *N mice* = 18, K108N *N mice* = 15). (**A**–**E**,**G**,**I**) Data are shown as mean ± SEM. (**F**,**H**,**J**) Data are shown as mean ± SD. * *p* < 0.05.

During the learning phase, comparison of the %CR, FEC, CRonset time and CRmaxA time between WT and K108N mice for CS trials showed no significant differences (Figure 4A–D). However, analysis of paired trials revealed a significantly different CRmaxA time between WT and K108N mice (time x condition, *p* = 0.01, F (9, 269) = 2.48; Supplementary Figure S5D), while the %CR, FEC and CRonset time did not differ (Supplementary Figure S5A–C). Pairwise comparison for paired trials on individual days did not yield a significant difference in CRmaxA time between conditions (Supplementary Figure S5D). During the memory retention-1 phase, the %CR for CS trials was significantly different between WT and K108N mice (time x condition, *p* = 0.005, F (3, 92) = 4.62; Figure 4A), while the FEC, the CRonset time and the CRmaxA time did not differ. Pairwise comparison for CS trials on individual days revealed a trend towards an increased %CR in K108N mice when compared to WT mice on D15 (*p* = 0.077, 95% CI = [−40.67, 1.53], Figure 4A). During the relearning phase, similarly to the learning phase, no significant differences were found between WT and K108N mice across any of the measured parameters for both CS and paired trials (Figure 4A–D; Supplementary Figure S5A–D). During the memory retention-2 phase, the %CR, FEC, CRonset time and CRmaxA time for CS trials did not significantly differ between WT and K108N mice (Figure 4A–D).

When examining the %CR in the transition phases for CS trials in WT and K108N mice, a significantly different %CR was found between conditions for the first transition (D12/15). WT mice showed a decrease in %CR from ~73% to ~65%, whereas K108N mice exhibited a slight increase in %CR from ~80% to ~84% (time x condition, *p* = 0.047, F (1, 33) = 4.27; Figure 4E). Pairwise comparison on individual days showed significantly increased %CR for K108N mice when compared to WT mice on D15 (*p* = 0.022, 95% CI = [−36.66, −2.49]; Figure 4E). Measuring the Δ %CR in each mouse between D12 and D15 confirmed the results for the %CR in the first transition (D12/15). Indeed, K108N mice performed significantly better than WT mice over the D12/15 transition (~+12%; *p* = 0.047, t = 2.07, df = 33; Figure 4F). No significant differences in the %CR or Δ %CR were found between conditions in the second (D25 ret/relrn) and third (relearning to memory retention-2, D26/29) transitions (Figure 4G–J).

In summary, these results show that genetically interfering with Sema3A signalling affects the beginning of the memory retention phase following the learning phase, suggesting that Sema3A may subtly limit the consolidation or expression of conditioned eyeblink memory.

### 2.5. Genetically Interfering with Sema3A Signalling Does Not Affect Structural Plasticity in the AIN After the Memory Retention Phase Following the Relearning Phase

To elucidate whether structural changes in the AIN of K108N mice may represent the anatomical substrate of the improved memory retention, we quantified the size and density of glutamatergic and GABAergic terminals in the AIN of mice at the end of the EBC protocol. We focused our analysis on the ipsilateral side of the US, namely the left AIN, i.e., the side in which greater plasticity is expected [53].

**Figure 5:**
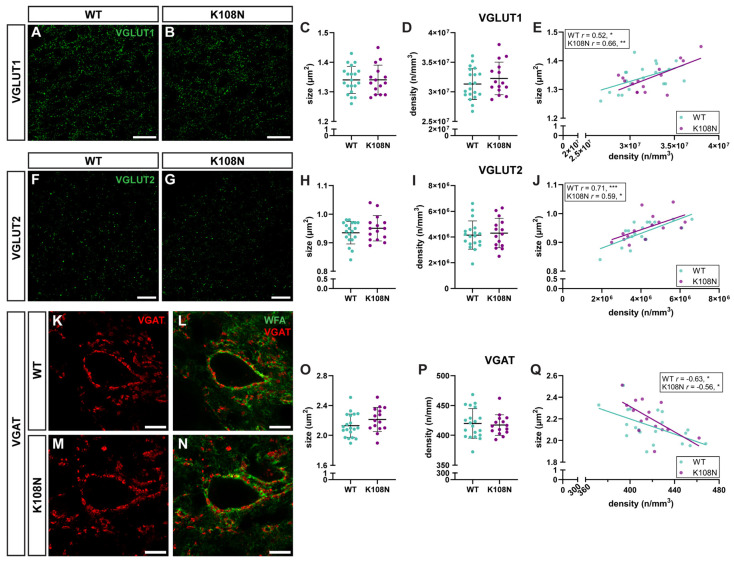
Genetically interfering with Sema3A signalling does not affect structural plasticity in the AIN after the memory retention phase following the relearning phase. Analysis was focused on the AIN ipsilateral to the US. (**A**,**B**) Representative images of VGLUT1^+^ terminals in WT (**A**) and K108N (**B**) mice. (**C**, **D**) Size (**C**) and density (**D**) of VGLUT1^+^ terminals in WT and K108N mice (WT *N mice* = 20, K108N *N mice* = 15). (**E**) Correlation matrix between size and density of VGLUT1^+^ terminals within WT and K108N mice (WT *N mice* = 20, K108N *N mice* = 15). (**F**,**G**) Representative images of VGLUT2^+^ terminals in WT (**F**) and K108N (**G**) mice. (**H**,**I**) Size (**H**) and density (**I**) of VGLUT2^+^ terminals in WT and K108N mice (WT *N mice* = 19, K108N *N mice* = 15). (**J**) Correlation matrix between size and density of VGLUT2^+^ terminals within WT and K108N mice (WT *N mice* = 19, K108N *N mice* = 15). (**K**–**N**) Representative images of VGAT^+^ terminals in WT (**K**,**L**) and K108N (**M**,**N**) mice. Analysis of VGAT^+^ terminals was conducted on WFA^+^ neurons (**L**,**N**). (**O**,**P**) Size (**O**) and density (**P**) of VGAT^+^ terminals in WT and K108N mice (WT *N mice* = 20, K108N *N mice* = 15). (**Q**) Correlation matrix between size and density of VGAT ^+^ terminals within WT and K108N mice (WT *N mice* = 20, K108N *N mice* = 15). Scale bar: (**A**,**B**,**F**,**G**): 40 µm; (**K**–**N)**: 10 µm. Data are shown as mean ± SD. Correlation coefficients (*r*) are indicated for each correlation matrix. * *p* < 0.05, ** *p* < 0.01, *** *p* < 0.001.

Glutamatergic terminals were visualised using anti-VGLUT1 and anti-VGLUT2 antibodies. VGLUT1 is expressed in terminals of a subset of mossy fibres, whereas VGLUT2 is present in terminals of inferior olive axons and a subset of mossy fibres [54]. In both genotypes, the size and density of VGLUT1^+^ terminals were greater than those of VGLUT2^+^ terminals (mean size, WT: VGLUT1, 1.34 µm^2^, VGLUT2, 0.94 µm^2^, K108N: VGLUT1, 1.34 µm^2^, VGLUT2, 0.95 µm^2^; mean density, WT: VGLUT1, 3.13 × 10^7^ n/mm^3^, VGLUT2, 4.15 × 10^6^ n/mm^3^, K108N: VGLUT1, 3.23 × 10^7^ n/mm^3^, VGLUT2, 4.31 × 10^6^ n/mm^3^; Figure 5C,D,H,I), which confirms previously published results [55,56]. Following EBC, VGLUT1^+^ terminals have been reported to be increased in the AIN of conditioned mice when compared to pseudo-conditioned mice [51]. No difference was found when comparing the size and density of VGLUT1^+^ or VGLUT2^+^ terminals in WT and K108N mice (Figure 5C,D,H,I). In addition, a positive correlation between the size and the density of VGLUT1^+^ terminals was found in both WT and K108N mice (Pearson’s correlation, WT: *r* = 0.52, *p* = 0.018, K108N: *r* = 0.66, *p* = 0.007; linear regression, WT: R^2^ = 0.27, *p* = 0.018, K108N: R^2^ = 0.44, *p* = 0.007; Figure 5E). A similar positive correlation was found between the size and the density of VGLUT2^+^ terminals in each condition (Pearson’s correlation, WT: *r* = 0.71, *p* = 0.0007, K108N: *r* = 0.59, *p* = 0.02; linear regression, WT: R^2^ = 0.50, *p* = 0.0007, K108N: R^2^ = 0.35, *p* = 0.02; Figure 5J). This indicates that mice with a higher density of excitatory terminals also have larger terminals. This demonstrates substantial variability in excitatory synaptic states in WT and K108N mice, which could lead to variations in EBC performance based on the synaptic state of each mouse. Thus, we correlated the size or the density of VGLUT1^+^ and VGLUT2^+^ terminals with EBC performance for each condition. No significant correlations between the size or the density of VGLUT1^+^ and VGLUT2^+^ terminals and the %CR on D15 (the day for which a significant increase in the %CR was found for K108N mice when compared to WT mice), or the Δ %CR of the first transition phase (D12/D15), were found in WT or K108N mice (Supplementary Figure S6A–H).

GABAergic terminals in the AIN, which mainly belong to Purkinje cells [57], were detected by anti-VGAT antibodies. The analysis of VGAT^+^ terminals was restricted to the somata of AIN neurons surrounded by a WFA^+^ PNN (Figure 5K–N). Similar to glutamatergic terminals, the size and density of VGAT^+^ terminals did not differ between WT and K108N mice (Figure 5O,P). Correlating the size and the density of VGAT^+^ terminals in each condition revealed a negative correlation (Pearson’s correlation, WT: *r* = −0.56, *p* = 0.011, K108N: *r* = −0.63, *p* = 0.012; linear regression, WT: R^2^ = 0.31, *p* = 0.011, K108N: R^2^ = 0.39, *p* = 0.013; Figure 5Q). This indicates that mice with an increased density of perisomatic inhibitory terminals have smaller terminals. This demonstrates substantial variability of GABAergic connectivity in WT and K108N mice, which could lead to variations in EBC performance based on the GABAergic connectivity of each mouse. Thus, we correlated the size or the density of VGAT^+^ terminals with EBC performance for each condition. Similar to glutamatergic terminals, no significance was reached when correlating either the size or density of VGAT^+^ terminals with the %CR on D15 or the Δ %CR of the first transition phase (D12/15) in WT or K108N mice (Supplementary Figure S6I–L).

### 2.6. Genetically Interfering with Sema3A Signalling Increases PNN-Associated Sema3A Intensity Around AIN Neurons After the Memory Retention Phase Following the Relearning Phase

**Figure 6:**
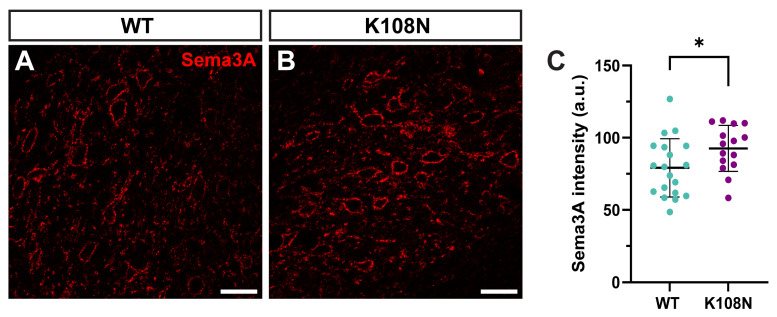
Genetically interfering with Sema3A signalling increases PNN-associated Sema3A intensity around AIN neurons after the memory retention phase following the relearning phase. Analysis was focused on the AIN ipsilateral to the US. (**A**,**B**) Example of Sema3A staining in the AIN of WT (**A**) and K108N (**B**) mice. (**C**) Quantification of Sema3A intensity in PNNs in WT and K108N mice (WT *N mice* = 19, K108N *N mice* = 15). Scale bar: (**A**,**B**): 40 µm. Data are shown as mean ± SD. * *p* < 0.05.

Sema3A in PNN^+^ CN neurons is reduced under conditions associated with plasticity, such as following enriched environmental stimulation [41]. The K108N mutation in Sema3A does not affect the ability of Sema3A to bind to the NRP1 receptor, but weakens its interaction with plexinA1/4, leading to impaired signalling [50]. However, it remains unclear whether this altered signalling affects Sema3A binding to PNNs. Thus, we analysed Sema3A signal intensity in PNNs of AIN neurons in WT and K108N mice at the end of the EBC protocol. Sema3A intensity was found to be significantly higher in PNNs surrounding AIN neurons of K108N mice when compared to WT mice (*p* = 0.043, t = 2.11, df = 32; Figure 6A–C).

## 3. Discussion

The aim of this study was to investigate the role of PNN-associated Sema3A in EBC learning and memory. To this end, we employed two approaches to interfere with Sema3A signalling: a local molecular approach and a global genetic approach. Disrupting Sema3A signalling in adult mice via molecular interference using NRP1 receptor bodies resulted in a decrease in EBC performance at the beginning of the memory retention phase and a decreased density of gephyrin puncta along the somatic membrane of AIN neurons after the relearning phase. However, the interpretation of these behavioural and histological outcomes is complicated by the inflammation observed at the injection sites (left and right AIN) induced by NRP1 receptor bodies. As the extent to which this inflammation contributed to the observed deficits cannot be determined, the specific role of Sema3A signalling disruption under these conditions remains uncertain. Interfering with Sema3A signalling through a global genetic approach using K108N mice did not lead to changes in the size or density of VGLUT1^+^, VGLUT2^+^, and VGAT^+^ terminals in the AIN at the end of the EBC protocol and did not exert a significant effect on EBC learning. However, it increased the immunohistochemical signal of Sema3A in PNNs surrounding AIN neurons at the end of the EBC protocol, and it improved EBC performance at the beginning of the memory retention phase.

Consistent with previous studies: (i) mice subjected to EBC learned to adjust the timing of their eyeblink when a CS was given so that the CR max amplitude time aligned with the US onset time [58,59,60,61,62,63], although the level of timing precision reported in the literature was not reached; (ii) CR onset time shifted over sessions towards the CS onset time for CS-only trials while it remained stable for paired trials, which is partially in line with previous research that found constant CR onset time throughout sessions [64,65]; and (iii) mice relearned faster following extinction (memory retention phase) when compared to the initial learning [66,67,68]. Taken together, these observations validate our EBC paradigm and setup, supporting the reliability of our EBC results.

### 3.1. Interfering with Sema3A Using a Local Molecular Approach

A reduction in %CR was found in NRP1 mice at the beginning of the memory retention phase. However, it remains unclear to what extent the inflammation found in the AIN of NRP1 mice disrupted the normal functioning of the AIN circuitry and thereby impaired EBC performance. Previous research reported that intracerebellar injection of tumour necrosis factor-α (TNF-α), a pro-inflammatory cytokine secreted by activated microglia, worsens EBC learning, likely due to impaired axonal conduction and/or glutamatergic neurotoxicity [69]. In vitro, acute application of TNF-α downregulates cell-surface GABA_A_ receptors, leading to reduced gephyrin clustering at the post-synaptic membrane [70]. Therefore, it is possible that TNF-α expression was induced in activated microglia in NRP1 mice, which in turn might have interfered with AIN neuron electrophysiology and EBC performance and induced a decrease in gephyrin density along the somatic membrane of AIN neurons. It is important to note that in NRP1 mice, only the beginning of the memory retention phase was affected, while after intracerebellar injection of TNF-α, the learning phase was impaired [69]. Thus, it is also possible that the EBC and gephyrin density changes observed in NRP1 mice are not consequences of activated microglia, but simply of Sema3A interference on cerebellar plasticity using NRP1 receptor bodies. Overall, the local molecular approach does not allow definitive conclusions regarding the role of Sema3A in cerebellum-dependent motor associative learning and memory.

### 3.2. What Is the Origin of the Inflammation?

Local AAV-NRP1 injections unexpectedly induced inflammation, whereas AAV-Fc did not, indicating that the inflammatory response depends on NRP1 receptor bodies, whether bound or unbound to Sema3A. Three complementary mechanisms could account for this inflammatory response: (i) NRP1 receptor bodies may act as neoantigens recognised by the immune system; (ii) NRP1 receptor bodies bound to PNN-associated Sema3A may form immune-like complexes, which activate FcγR-mediated pro-inflammatory signalling in microglia via interaction with the Fc-domain of NRP1 receptor bodies; and (iii) Sema3A has a role in the regulation of inflammatory processes in the brain, and its sequestration by NRP1 receptor bodies disrupts this function.

Foreign transgene products expressed via, e.g., AAVs, can act as neoantigens in the brain. Their local production in glia cells can trigger antigen presentation and T-cell recruitment, which can cause a strong microglial activation around transduced cells [71]. Given that the NRP1 DNA sequence used in the NRP1 receptor body is of rat origin and that the fusion of the Fc-fragment to NRP1 results in novel epitopes, expression of the NRP1 receptor body may elicit microglial activation through immune recognition mechanisms.

Sema3A binds CSPGs within PNNs [16,17]. The putative PNN-binding domain of Sema3A is thought to reside in its C-terminal tail, based on amino acid sequence similarity to orthodenticle homeobox 2, a protein implicated in PNN binding [18,72]. The NRP1-binding domain is located within the Sema domain and the C-terminal tail, near the putative PNN-binding domain [9,18]. Consequently, NRP1 receptor bodies may interact with Sema3A already associated with CSPGs. In this configuration, immobilised NRP1 receptor bodies could mimic an immune complex, which may promote complement activation and the transition of microglia to a pro-inflammatory state [73]. Activated microglia upregulate a range of immune-related genes, including Fcγ receptors (FcγRI and FcγRIII) [74], which, upon clustering via immune complex binding, contribute to the induction of pro-inflammatory signalling pathways [75]. Thus, immune-like complexes involving immobilised NRP1 receptor bodies, through binding to PNN-associated Sema3A, may bind FcγRs, allowing FcγR crosslinking, and thereby induce FcγR-mediated pro-inflammatory signalling in local microglia.

Whether Sema3A-NRP1 signalling regulates immune cell function in the healthy brain is not known. However, once the inflammation is present, Sema3A and NRP1 receptors have established roles in modulating inflammation through interactions with a range of immune cell types in the periphery [76,77]. Some of these mechanisms may also operate within the central nervous system (CNS). For instance, Sema3A signalling via NRP1 receptors reduces T-cell proliferation under inflammatory conditions [76,77], a process that can occur in the CNS if the blood–brain barrier (BBB) is compromised. In a multiple sclerosis mouse model in which the BBB is altered, NRP1 receptors regulate CD4^+^ T-cell migration into the CNS [78]. However, it remains unclear whether the inflammation observed in NRP1 mice led to local disruption of the BBB. Activated microglia express NRP1 receptors [76,77]. In vitro, Sema3A induces NRP1-dependent apoptosis of activated microglia, a finding supported in vivo by observations that stressed neurons release Sema3A and that microglia upregulate NRP1 receptor expression following focal brain injury, allowing Sema3A to bind to NRP1 receptors expressed by microglia [79]. Thus, sequestration of Sema3A by NRP1 receptor bodies may block these regulatory interactions and thereby contribute to the persistence of the inflammation observed in NRP1 mice.

### 3.3. How to Improve NRP1 Receptor Bodies?

Over the past four decades, extensive research has focused on mutating the Fc domain to prevent its interaction with FcγRs [80]. Our unpublished observations suggest that replacing the Fc domain of the NRP1 receptor body with a less immunogenic mutated variant harbouring mutations at positions L234A, L235A, and P329G (LALAPG), which abolish FcγRs binding [80], and expressing it throughout the brain via systemic administration (AAV-PhP.eB) does not increase cortical Iba1 immunoreactivity. In the context of amyloid-β (Aβ) pathology, inserting the same LALAPG mutation into the Fc domain of Lecanemab—a monoclonal antibody that attenuates Aβ pathology by binding amyloid plaques—abolishes microglia-mediated amyloid clearance, indicating that Fc domains within Lecanemab-FcLALAPG:amyloid plaques immune complexes are no longer recognised by microglia FcγRs [81]. A similar mechanism likely explains our observations that improved NRP1-FcLALAPG receptor bodies do not trigger microglial activation. Although systemic administration of less immunogenic NRP1 receptor bodies using AAV-PhP.eB probably resulted in lower local protein concentrations, higher local viral load achieved through direct intraparenchymal injections is expected to yield comparable results. This approach would allow functional investigation of Sema3A in cerebellum-dependent motor associative learning and memory while minimising inflammation-related confounding effects.

### 3.4. Sema3A Function in Learning and Memory

Improved EBC performance at the beginning of the memory retention phase following the learning phase was found in K108N mice, suggesting that Sema3A subtly limits early memory consolidation or expression, leaving learning and relearning processes unaffected. The effect of Sema3A appears to be restricted to a specific time-dependent process or learning threshold, as the EBC performance enhancement found is not sustained throughout the memory retention phase, and is absent during the second memory retention phase. This finding expands the current knowledge on the role of Sema3A in learning and/or memory [41,82,83,84]. Following exposure to an enriched environment, a context known to improve learning and memory [85], perisomatic Sema3A levels are decreased in the AIN of mice [41]. Injection of an antibody binding to chondroitin 4-sulfates—a PNN sulfation pattern which inhibits neurite growth [86]—in the perirhinal cortex of mice improves long-term (1-day) object recognition memory [82]. This effect may involve interference with Sema3A signalling, as this antibody partially blocks Sema3A binding to PNNs and decreases PNN formation in vitro and in vivo [82]. Following passive avoidance fear conditioning, Sema3A levels are increased in the hippocampus of mice after a single presentation of an aversive stimulus [83]. Hippocampal injection of Sema3A-neutralising antibodies in rats prior to passive avoidance fear conditioning impairs learning and/or memory consolidation when animals are tested 30 min after a single presentation of an aversive stimulus [83]. Conversely, basolateral amygdala injection of Sema3A prior to cued-associative fear conditioning impairs memory consolidation 30 min after conditioning [84]. This suggests that Sema3A is rapidly involved in learning and/or memory consolidation processes. Additional EBC experiments including memory retention sessions on the same day as each learning session, preceding and/or following that learning session, would give more insights into the role of Sema3A in short-term learning and/or memory consolidation.

In the global genetic approach, Sema3A signalling is impaired throughout the brain from embryonic development onward, resulting in loss of Sema3A-mediated chemorepulsion for axon guidance and exuberant axonal growth [50]. It is therefore possible that the improved EBC performance observed in K108N mice results from developmental alterations in synaptic innervation involved in EBC, for instance, of Purkinje cells and AIN neurons. Thus, the observed EBC phenotype may originate from developmental modifications of input/output connections of Purkinje cells and/or AIN neurons, which are essential for EBC learning and memory [46,87]. Indeed, Sema3A is involved in the repulsion of mossy fibres, which project to AIN neurons and granule cells, as well as in the elimination of surplus climbing fibres, which project to Purkinje cells [88,89].

### 3.5. Sema3A Function in Plasticity

No changes in the size or density of VGLUT1^+^, VGLUT2^+^ or VGAT^+^ terminals in the AIN of K108N mice were observed after the memory retention phase following the relearning phase. This suggests that Sema3A is not involved in regulating remodelling of pre-synaptic terminals in the AIN following EBC. However, it may be that Sema3A does play a role in EBC-dependent AIN pre-synaptic structural plasticity, but not at the analysed time point where no behavioural differences were found. Analysing changes in pre-synaptic terminals after the beginning of the memory retention phase following the learning phase, where an improvement in EBC performance was found in K108N mice, may highlight differences between WT and K108N mice. Indeed, an increased density of VGLUT1^+^ and gephyrin^+^ terminals in the AIN has been observed following EBC learning in conditioned mice compared to pseudo-conditioned mice [51]. Thus, after the final memory retention phase, the EBC network formed during learning may be stabilised and no longer require additional VGLUT1^+^ and gephyrin^+^ terminals, i.e., further mossy fibre collateral and Purkinje cell axon formation. An increasing number of studies suggest a role for Sema3A in adult brain plasticity [42,83,90,91,92]. An increased density of VGLUT2^+^ terminals and an increased size of VGAT^+^ terminals were found in the AIN of mice following exposure to an enriched environment [42], in parallel with a decrease in perisomatic Sema3A [41]. In cultured hippocampal neurons, the addition of Sema3A decreases the size of synaptophysin and PSD-95 puncta, pre- and post-synaptic markers, respectively [90], and increases the number of AMPA receptor subunits GluA1 at the dendritic membrane [83]. In cultured cortical neurons, the addition of Sema3A promotes clustering of synapsin, a pre-synaptic marker, and PSD-95 [91]. Recreating a similar experiment for CN neurons is challenging, as pre-synaptic afferences to CN neurons originate from Purkinje cell axons as well as mossy-fibre and climbing-fibre collaterals, requiring the co-culture of these neuronal populations to ensure proper synaptic connectivity. However, organotypic cultures of cerebellar, pontine and inferior olivary slices combined with microfluidic compartmentalisation [93] to guide axonal growth could allow the study of Sema3A-dependent CN plasticity in vitro. Additionally, Sema3A appears to be involved in a universal pre-synaptic homeostatic plasticity (PHP) mechanism throughout the nervous system. At the neuromuscular junction (NMJ), PHP is absent in K108N mice but restored by adding Sema3A, while incubating WT NMJs with mutated Sema3A^K108N^ abolishes it [92]. The same pattern occurs in the hippocampus: PHP is absent in K108N mice, but restored by addition of Sema3A and abolished by Sema3A knockdown in WT mice or incubation of mutated Sema3A^K108N^ in WT hippocampal slices [92]. These findings suggest that Sema3A may contribute to PHP within the AIN, modulating synaptic output during early post-learning phases. The transient improvement observed at the beginning of the memory retention phase in K108N mice may be explained by reduced homeostatic regulation at synapses engaged during EBC learning, which may promote CRs. Whether this effect reflects altered early memory consolidation or expression remains to be determined.

### 3.6. Modulation of Perisomatic Sema3A

A slightly stronger Sema3A immunofluorescent signal in the perisomatic region of AIN neurons, where the PNN is localised, was detected in K108N mice after the memory retention phase following the relearning phase. This increase may be due to: (i) an increased synthesis of Sema3A protein to compensate for the reduced signalling; (ii) a change in Sema3A conformation due to the mutation, which may alter Sema3A processing/turnover; (iii) a change in Sema3A shape which may uncover additional epitopes and thus facilitate antibody binding during immunostaining. Regardless of the origin of this increase, this result demonstrates that the K108N mutation in Sema3A does not impair its ability to bind PNNs. This was expected, as this mutation is located in the Sema domain [50], whereas the putative PNN-binding domain is predicted to reside in the C-terminal tail, as described above [18,72].

### 3.7. Conclusions

Local expression of NRP1 receptor bodies in the AIN does not constitute an effective approach to investigating the role of Sema3A in cerebellum-dependent motor associative learning and memory, as it triggers inflammation. This limitation might be overcome by employing a less immunogenic variant of the NRP1 receptor body. Genetically interfering with Sema3A using the K108N mouse line represents a valid approach to investigating Sema3A function in cerebellum-dependent motor associative learning and memory. The enhanced EBC performance observed at the beginning of the memory retention phase following the learning phase in K108N mice suggests that constitutive Sema3A subtly limits early memory consolidation or expression. To determine whether a learning threshold is required for Sema3A to influence memory consolidation or expression, and to assess the timing of its action, additional memory retention sessions should be given prior to and after each learning session. Moreover, analysis of plasticity and perisomatic Sema3A levels in the AIN should be performed at the time point at which EBC performance of K108N mice improves in order to correlate it with the change in behaviour. However, the use of the K108N mouse line does not allow for discrimination between the developmental or adult effect of the Sema3A mutation on EBC performance. Thus, selective interference with Sema3A in adult subjects, such as by using the less immunogenic variant of the NRP1 receptor body, is required to exclude developmental contributions and to gain a clearer understanding of the adult Sema3A role in learning and memory processes.

Overall, due to an inflammatory phenotype detected in NRP1 mice, we cannot draw definitive conclusions on the behavioural and structural changes observed following adult-specific manipulation of Sema3A signalling. Nonetheless, our data based on K108N mice reveal that constitutively altered Sema3A signalling is associated with a subtle improvement in cerebellar memory. Future studies will have to elucidate the underlying mechanism of this association.

## 4. Materials and Methods

### 4.1. Animals

All experimental procedures involving animals were approved by the animal committee of the Royal Netherlands Academy of Arts and Sciences and adhered to the European guidelines for the care and use of laboratory animals (Council Directive 86/6009/EEC). Adult male, wild-type C57BL/6J mice from Janvier Labs (Le Genest-Saint-Isle, France; WT; *N* = 45), wild-type C57BL/6J mice bred in-house (WT; *N* = 20) and K108N homozygous mutant mice (K108N; *N* = 19) of around two and a half months of age were used for this study. The K108N mouse line [50] was provided by Prof. Ginty (Harvard Medical School, Boston, MA, USA), bred in-house, and rederived from a C3H/He-C57BL/6J mixed strain background to a C57BL/6J strain background (5 backcrossings). Mice were socially housed with ad libitum access to food and water and were kept on a normal light cycle (12 h:12 h light/dark). Experiments were performed in the daytime.

### 4.2. Pedestal Placement for Eyeblink Conditioning

Around 20 min prior to the start of the surgery, mice received a subcutaneous injection of carprofen (5 mg/kg; Rimadyl^®^, Zoetis, Parsippany-Troy Hills, NJ, USA). The mice were deeply anaesthetised using isoflurane anaesthesia (induction 4–5%, maintenance 1–2% in medicinal air), their heads were shaved and they were placed on a stereotaxic apparatus with a heating pad and a rectal probe to keep the body temperature at 37 °C. Their eyes were covered with ointment and paper to prevent corneal dryness and retinal damage. The head skin was cleaned with hibicet and locally anaesthetised with a lidocaine spray (100 mg/mL; Xylocaine^®^ spray, AstraZeneca, Cambridge, UK). A 1 cm midline sagittal skin incision was made on the skull, and lidocaine cream (50 mg/g; Xylocaine^®^ cream, AstraZeneca, Cambridge, UK) was spread on the incision to locally anaesthetise the periosteum. Subsequently, the skull surface was cleaned with saline and dried. An etching component (#36517, OptiBond^TM^ Universal Kit, Kerr, Kloten, Switzerland) was applied on the skull and cleaned with saline after 10–15 s. Primer (#36517, OptiBond^TM^ Universal Kit, Kerr, Kloten, Switzerland) was then applied on the skull and hardened using UV light. A small aluminium block (6 × 3 × 5 mm; “pedestal”) was stereotaxically placed on top of the primer and fixed with dental cement (#595953WW, Tetric EvoFlow^®^ A1, Ivoclar, Schaan, Liechtenstein), which was also hardened using UV light. The skin was then pulled over the dental cement, and tissue glue (#1469SB, Vetbond^TM^, Maplewood, MN, USA) was applied. Afterwards, animals were placed in a heated chamber until they recovered from anaesthesia. The day after surgery, drinking water with 0.06 mg/mL of carprofen was given to the mice for 3 consecutive days.

### 4.3. Viral Vector Injections

C57BL/6J mice ordered from Janvier received a bilateral injection of adeno-associated viral vectors (AAVs) in the AIN prior to pedestal placement. Mice were injected 3 weeks prior to the start of the learning phase, resulting in an age of approximately 2.5 months at learning onset (see Section 4.5 below). AAVs were produced as previously described [94]. The design, expression and functional characterisation of AAV8-NRP1(Y297A)-Fc and AAV8-Fc under a cytomegalovirus (CMV) promoter were reported by Boggio and collaborators [49]. Mice were prepared as described in Section 4.2. Mice received a subcutaneous injection of buprenorphine (0.1 mg/kg; Bupaq^®^, Vetviva, Wels, Austria) pre-operatively in addition to carprofen due to the increased invasiveness of the surgery. A 1.5 cm midline sagittal skin incision was made, giving access to the interparietal bone of the skull. Two small craniotomies were drilled bilaterally, and 2 μL of AAV8-CMV-Fc-WPRE (Fc group; 2E12GC/mouse) or AAV8-CMV-NRP1(Y297A)-Fc-WPRE (NRP1 group; 2.4E12GC/mouse) was injected in each side (A/P: −2 to −2.2 mm from lambda, Lat: ±1.7 to 1.8 mm, D/V: −2.2 to −2.3 mm) by using a quartz capillary pipette (30–40 μm tip diameter) connected to a Harvard injection pump at a rate of 0.2 μL/min. The capillary was left in place for 5 min post-injection and then slowly retracted. The skull preparation and pedestal placement procedure were performed as described in Section 4.2, and the head skin was closed using resorbable sutures and tissue glue. Post-surgery care was performed as described in Section 4.2.

### 4.4. Eyeblink Conditioning Setup

The eyeblink conditioning setup consists of a head-fixed mouse, attached via its pedestal on a freely rotating cylindrical treadmill, placed inside a soundproof, light-free box (Neurasmus B. V., Erasmus MC, Rotterdam, The Netherlands). The mouse was constantly monitored via an infrared camera present in the box. A solenoid valve (#INKA1224212H, The Lee Company, Westbrook, CT, USA) connected to a pressure controller (#R07-200-RNKG, IMI plc, Birmingham, UK) was perpendicularly placed in front of the left eye to give a corneal air puff (~30 psi) [unconditioned stimulus (US)]. A module composed of 4 green LED lights (3.5 mm in diameter), placed in a square and spaced by 3 mm, was placed ~6 cm in front of the mouse to present a flash of light [conditioned stimulus (CS)]. All 4 LEDs present on the module were flashing concomitantly. The delivery of the puff as well as the LED flash was executed using custom-written LabVIEW software (LabVIEW 2022 Q3, National Instruments, Austin, TX, USA) in combination with National Instruments hardware (National Instruments, Austin, TX, USA) and Neurasmus hardware (Neurasmus B. V., Erasmus MC, Rotterdam, The Netherlands). Eyelid movements of the left eye were recorded using a high-speed infrared camera (751 fps, acA640–750 um, Basler AG, Ahrensburg, Germany) in combination with an infrared light and converted in real time into traces using custom-written LabVIEW software (National Instruments, Austin, TX, USA). A rectangular region of interest (ROI) of ~20 × 280 pixels was drawn to include the longest distance between the upper and lower eyelids, while excluding the infrared light reflection. For each frame, a Butterworth filter was applied, and the greyscale value of each pixel within the ROI at a given frame was averaged (pixel value ranging from 0 to 255). This average represents the fraction of eyelid closure (FEC).

### 4.5. Eyeblink Conditioning Protocol

A total of 7 WT, 19 Fc and 19 NRP1 mice for the local molecular approach and 20 WT bred in-house and 19 K108N mice for the global genetic approach underwent the following EBC protocol. Mice were first handled for 5 days. Then, mice were habituated in the box for 2 days by being fixed on the freely rotating cylindrical treadmill for ~30 min, after which the following protocol was presented: 2 blocks composed of 1 US (30 ms) and 10 CS (280 ms each). Mice were then trained for 10 days (2 times for 5 consecutive days with a 2-day break in between, days 1–5 and 8–12; ‘learning phase’) with the following protocol for each training session (1 session/day): 20 blocks composed of 1 US, 10 CSUS (paired trials) and 1 CS. A paired trial consists of a CS and a US, starting with a 250 ms delay, which co-terminate. Each trial was separated by a randomised intertrial interval of 8 s ± 3 s to avoid associative learning based on timing. At the start of the training, the distance between the air puff and the mouse’s eye was set at ~0.5 cm. If needed, this distance was adjusted during a training session (maximum 3 adjustments per session) and throughout the training in order to obtain an optimal blink. Mice subsequently proceeded to the ‘memory retention phase’, during which 4 retention sessions were given on days 15, 19, 22 and 25. The memory retention protocol consisted of 1 US and 10 CS. During this phase, the distance between the air puff and the mouse’s eye was kept at ~0.5 cm. Subsequently, mice entered the ‘relearning phase’ (day 25 and 26), where the same protocol as the ‘learning phase’ was presented, including the setup and adjustment of the distance between the air puff and the mouse’s eye. An additional memory retention day was added for WT and K108N mice with the same parameters as described above (day 29). Custom-written LabVIEW software (National Instruments, Austin, TX, USA) in combination with National Instruments hardware (National Instruments, Austin, TX, USA) and Neurasmus hardware (Neurasmus B. V., Erasmus MC, Rotterdam, The Netherlands) was used to execute each protocol and record each session.

### 4.6. Analysis of Eyeblink Conditioning Data

The eyelid traces were analysed offline using custom-written MATLAB scripts (version R2025a, MathWorks, Natick, MA, USA). The following temporal windows were used for each trial: 0–500 ms baseline, 500–780 ms CS (CS and paired trials), 750–780 ms US (US and paired trials), 780–2000 ms post-stimuli period. First, eyelid traces were normalised to their respective baselines. Eyelid traces with an unstable baseline were removed (variation exceeding 3 times the standard deviation during the baseline period). Subsequently, minimum (mEC) and maximum (MEC) eyelid closure values of US trials were extracted. During the learning phase, substantial within-session variability was observed in baseline amplitudes across trials, likely due to eye discomfort caused by repeated corneal puffs. Thus, to minimise the impact of this variability, all stimuli within a session were divided into 5 consecutive groups (blocks 1–4, 5–8, etc.). Within each group, the mEC values of US trials were subtracted from their corresponding MEC values (corrected MEC), corrected MEC values were averaged, and trial types (US, paired and CS) were normalised to this average. During the memory retention phase, US and CS were normalised to the corrected MEC value of the single US trial. Trials for which the eyelid closure exceeded 10% of the MEC within 560–850 ms for CS trials and 560–750 ms for paired trials were considered CRs. Eyelid closure exceeding 10% of the MEC within 500–560 ms for CS and paired trials was excluded, as only a startle response can occur during this time frame [95]. For each session, the percentage of CR (%CR), the normalised FEC, ranging from 0 to 1 (eyelid fully open or closed, respectively), the averaged onset time of CRs (CRonset time), and the averaged MEC time of CRs (CRmaxA time) were calculated. These parameters were measured for CS and paired trials. Mice that never reached a %CR higher than 10% for at least 2 days during the ‘learning phase’ were considered non-learners and thus excluded from the analysis. Mice that mistakenly underwent an extra session were included in the analysis; however, only sessions acquired prior to the error were used. This includes 1 NRP1 mouse excluded after the ‘learning phase’, 1 NRP1 mouse excluded after the ‘memory retention phase’, and 2 K108N mice excluded after the ‘memory retention-1 phase’. For two NRP1 mice, the final memory retention session was excluded due to an unclean US. However, subsequent sessions were retained as the memory retention procedure was correctly performed but could not be analysed due to the unclean US. Additionally, EBC performance Δ of %CR (Δ %CR) for CS trials of each transition phase was quantified by dividing the %CR on the last day of one phase by that on the first day of the following phase. Mice for which one of the sessions was missing were excluded from the corresponding transition analysis but retained for all other analyses (e.g., a mouse could be retained for the D12/15 transition analysis but excluded from the D25 ret/relrn transition analysis). This includes 1 NRP1 mouse for the D12/15 transition and D25 ret/relrn transition, 3 NRP1 mice for the D25 ret/relrn transition, and 2 K108N mice for the D25 ret/relrn transition and D26/29 transition.

### 4.7. Histology

Following the last EBC session, mice were anaesthetised via an intraperitoneal injection of an overdose of pentobarbital (Euthasol^®^, Apotheek Universiteit Utrecht, Utrecht, The Netherlands) (500 mg/mL) and transcardially perfused with ~30 mL of freshly prepared phosphate-buffered saline (PBS, pH 7.4) followed by ~85 mL of freshly prepared 4% paraformaldehyde (PFA) in phosphate buffer (PB, 0.1 M, pH 7.4). Brains were post-fixed overnight in 4% PFA and cryoprotected in 0.1 M PB containing 30% sucrose at 4 °C. If the tissue was not sectioned within 2 weeks, the solution was replaced by a 30% sucrose solution with 0.02% Na-azide for longer preservation. Cerebella were cut in 25 µm thick sagittal or coronal floating slices using a freezing sledge microtome (Hyrax S30, Zeiss, Oberkochen, Germany) or a cryostat (CM3050S, Leica, Wetzlar, Germany). Slices were stored in a PBS solution with 0.02% Na-azide and kept at 4 °C for long-term storage. Staining was performed as follows: Slices were incubated overnight at 4 °C with primary antibodies and 5% foetal calf serum (FCS) diluted in PBS with 0.25% Triton X-100 (PBST). Then slices were incubated for 2 h at room temperature (RT) with secondary antibodies and 2.5% FCS diluted in PBST, followed by an incubation for 5 min at RT with DAPI (1:10,000, #D9542, MilliporeSigma, Burlington, MA, USA) diluted in PBST. Between each step, slices were washed for 10 min in PBST (3 times). The primary antibodies used were: rabbit anti-Fc (1:1000, #31142, ThermoFisher, Waltham, MA, USA); guinea pig anti-Iba1 (1:500, #234 308, Synaptic Systems, Goettingen, Germany); mouse anti-gephyrin (1:500, #147 021, Synaptic Systems, Goettingen, Germany); guinea pig anti-vglut1 (1:500, #135 304, Synaptic Systems, Goettingen, Germany); rabbit anti-vgat (1:1000, #131 002, Synaptic Systems, Goettingen, Germany); biotinylated WFA (1:200, #B-1355, Vector Laboratories, Newark, CA, USA). The secondary antibodies used were: donkey anti-rabbit-488 (#711-546-152, Jackson Immunoresearch, West Grove, PA, USA); donkey anti-guinea pig-488 (#706-545-148, Jackson Immunoresearch, West Grove, PA, USA); donkey anti-guinea pig-Cy3 (#706-165-148, Jackson Immunoresearch, West Grove, PA, USA); goat anti-mouse IgG1-Cy3 (#115-165-205, Jackson Immunoresearch, West Grove, PA, USA); donkey anti-guinea pig-647 (#706-605-148, Jackson Immunoresearch, West Grove, PA, USA); streptavidin-Cy3 (#016-160-084, Jackson Immunoresearch, West Grove, PA, USA). Dilution for secondary antibodies was kept standard at 1:500 from an antibody stock diluted 1:1 in glycerol. Fc staining was performed prior to other staining of the same tissue to prevent cross-reactivity of the Fc primary antibody with the Fc regions of other primary antibodies.

For Sema3A immunofluorescence, slices were first washed 5–6 times for 10 min in PBS to discard the PBS solution with 0.02% Na-azide (storing solution). Next, slices were incubated with chondroitinase ABC (ChABC, 0.1 U/mL; #C3667, MilliporeSigma, Burlington, MA, USA) for 2 h at 37 °C in ChABC buffer (0.1 M Tris–HCl, 0.03 M sodium acetate, pH 8.0) to partially digest the PNN, as this step significantly improves the signal intensity of Sema3A in the PNN [11]. Then, slices were processed with a tyramide signal amplification biotin (TSA-B) kit (#NEL700A001KT, Akoya, Marlborough, MA, USA) following the manufacturer’s protocol. Next, slices were incubated overnight at RT with a goat anti-Sema3A antibody (1:250, #sc-1146, Santa Cruz Biotechnology, Dallas, TX, USA). The following day, slices were incubated with a secondary biotinylated horse anti-goat IgG antibody (1:200, #BA-9500-1.5, Vector Laboratories, Newark, CA, USA) for 30 min at RT, followed by an incubation with streptavidin-HRP (1:150, TSA-B kit) for 30 min at RT. Slices were then incubated with biotinylated tyramide (1:60, TSA-B kit) for 9 min at RT. Lastly, slices were incubated with streptavidin-Cy3 (#016-160-084, Jackson Immunoresearch, West Grove, PA, USA) diluted in PBS for 30 min at RT. Staining for other markers was performed on the same tissue following the classical staining protocol described above. After each step of Sema3A staining, slices were washed 3 times for 10 min in PBS. Finally, the sections were mounted in Mowiol (#4759045-M, MilliporeSigma, Burlington, MA, USA).

### 4.8. Quantification of Gephyrin Terminals

Images were acquired using a confocal microscope (Abberior STEDYCON, Gottingen, Germany, coupled to a ZEISS AxioVert 200M, Oberkochen, Germany), a 100× oil objective (NA 1.46, pinhole 50 µm), a pixel dwell time of 3 µs with 4 lines of accumulation, and a resolution of 100 nm^2^ pixel size. The density of gephyrin puncta in the perisomatic region of neurons within the anterior interposed nucleus (AIN; left and right hemispheres), which were either Fc^+^ or in close proximity to Fc^+^ neurons, was measured in Fc and NRP1 mice using Fiji software (version 1.53t) [96] (~15 neurons/side/mouse from an average of 3 slices per side; left and right hemispheres merged: Fc *N mice* = 11, NRP1 *N mice* = 16; left and right hemispheres separately: Fc *N mice* = 11, NRP1 *N mice* = 13). Three NRP1 mice were not included in the separated hemispheres analysis due to an unclear distinction between the two sides. In Fiji software, first, the lowest 40% of pixel values were averaged per image (1 neuron/image). Then, the values from all images were averaged to obtain the background intensity for each side per mouse. Finally, a line profile was drawn in the perisomatic region of neurons, and the density of gephyrin puncta (number of terminals/mm) was manually calculated and averaged per mouse. Positive gephyrin puncta were defined as peaks above the threshold value (3 times the background intensity) in the line profile.

### 4.9. Quantification of Fc and Iba1

Images were acquired using a slide scanner microscope (ZEISS Axioscan 7, Oberkochen, Germany) under a 10× objective, an exposure time of 150 ms for Fc and 200 ms for Iba1, and a resolution of 0.65 µm^2^ pixel size. The intensity of Fc and Iba1 staining was measured in Fc and NRP1 mice by drawing in both hemispheres a region of interest (ROI) around the AIN by using Qupath software (version 0.4.3) [97] (3 slices/mouse; hemispheres merged, Fc *N mice* = 19, NRP1 *N mice* = 19; left hemisphere, Fc: Fc *N mice* = 18, NRP1 *N mice* = 14, Iba1: Fc *N mice* = 18, NRP1 *N mice* = 15; right hemisphere, Fc and Iba1: Fc *N mice* = 18, NRP1 *N mice* = 15). One Fc mouse and four NRP1 mice were not included in the separated hemispheres analysis due to an unclear distinction between the two sides. One additional NRP1 mouse was removed from the Fc analysis in the left hemisphere due to abnormal staining. For each slice, the background intensity was obtained by drawing an ROI in an Fc negative cellular nucleus or between Iba1^+^ cells in the AIN, and the average of the background values of all slices was calculated for each mouse. This average was subtracted from the Fc or Iba1 intensity of the same mouse. Iba1 staining value of each slice was then averaged per mouse. Zoom images were acquired using a confocal microscope (Leica TCS SP5, Wetzlar, Germany), a 40× oil objective (NA 1.3, pinhole 65 µm), an acquisition speed of 100 Hz, and a resolution of 189 nm^2^ pixel size.

### 4.10. Quantification of VGLUT1 and VGLUT2 Terminals

VGLUT1 images were acquired using a confocal microscope (Abberior STEDYCON, Oberkochen, Germany coupled to a ZEISS AxioVert 200M, Oberkochen, Germany), a 40× oil objective (NA 1.3, pinhole 25 µm), a pixel dwell time of 3µs with 4 lines of accumulation, and a resolution of 125 nm^2^ pixel size. VGLUT2 images were acquired using a confocal microscope (Leica TCS SP5), a 40× oil objective (NA 1.3, pinhole 65 µm), an acquisition speed of 50 Hz, and a resolution of 154 nm^2^ pixel size. The size and density of VGLUT1 and VGLUT2 puncta within the AIN (left hemisphere) were measured in WT and K108N mice (~3 slices/mouse; VGLUT1, WT *N mice* = 20, K108N *N mice* = 15; VGLUT2, WT *N mice* = 19, K108N *N mice* = 15). A Grubbs’s test revealed 1 WT outlier from VGLUT2 data, which was then removed (α = 0.05, G = 3.48). Images were taken in the centre of the AIN, at a similar medio-lateral position for each slice. Two images per ROI, with a step size of 0.5 µm, were taken. A custom-written Fiji software macro (version 1.54p) was used to analyse the data in a fully automated approach. Each image was pre-processed using the following steps: maximum intensity z-projection, convert to 8-bit, auto threshold, convert to mask, watershed. The “analyse particle” function was used to estimate the size (µm^2^) and the density (number of terminals/mm^2^) of glutamatergic boutons. Based on trial and error and the literature, only particles with a size between 0.4 μm^2^ and 5 μm^2^ were included in the analysis [55,56]. To convert the density to mm^3^, the optical section thickness was calculated for each acquisition setting by measuring its respective axial resolution. Finally, the size and density of VGLUT1 and VGLUT2 puncta per slice were averaged per mouse.

### 4.11. Quantification of VGAT Terminals

Images were acquired using a confocal microscope (Abberior STEDYCON coupled to a ZEISS AxioVert 200M), a 100× oil objective (NA 1.46, pinhole 50 µm), a pixel dwell time of 3µs with 4 lines of accumulation, and a resolution of 80 nm^2^ pixel size. The size and density of VGAT puncta in the perisomatic region of WFA^+^ neurons within the AIN (left hemisphere) were measured in WT and K108N mice (~15 neurons/mouse from an average of 3 slices; WT mice: N = 20; K108N mice: N = 15). Two images per ROI, with a step size of 0.3 µm, were taken. A custom-written Fiji software macro (version 1.54p) was used to analyse the data in a semi-automated approach. Each image was pre-processed using the following steps: maximum intensity z-projection, subtract background, enhance contrast, convert to 8-bit, auto threshold, convert to mask, adjustable watershed. An ROI including all GABAergic boutons in the perisomatic region of an AIN WFA^+^ neuron was drawn. In addition, a line was drawn around the edge of the same neuron to define the perimeter. The “analyse particle” function was used to estimate the size (µm^2^) and the density (number of terminals/mm) of GABAergic boutons. Manual correction of the selected particles from the “analyse particle” function was made if required (e.g., 2 particles selected when there should be 1). Only particles with a size between 0.5 μm^2^ and 10.75 μm^2^ were included in the analysis [98]. Finally, the size and density of VGAT puncta were averaged per neuron, then per mouse.

### 4.12. Quantification of Sema3A^+^ PNNs

Images were acquired using a confocal microscope (Leica TCS SP5), a 40× oil objective (NA 1.3, pinhole 65 µm), an acquisition speed of 50 Hz, and a resolution of 154 nm^2^ pixel size. The intensity of Sema3A staining around neurons in the AIN (left hemisphere) was measured in WT and K108N mice (3 slices/mouse; WT *N mice* = 19, K108N *N mice* = 15). Images were taken in the centre of the AIN, at a similar medio-lateral position for each slice. Using Fiji software (version 1.53t), ROIs enwrapping each Sema3A^+^ PNN were drawn. A background intensity per slice was obtained by drawing a single ROI inside 3 Sema3A^+^ neurons, and the background average of these neurons was subtracted from the Sema3A intensity of the same slice. Finally, Sema3A slice intensities were averaged per mouse.

### 4.13. Statistics

Statistics were performed using GraphPad Prism 10 (version 10.4.1). The normality of the data was tested using the D’Agostino and Pearson test, Anderson–Darling test, Shapiro–Wilk test or Kolmogorov–Smirnov test. If normality was violated, non-parametric tests were used. EBC parameters across the learning, memory retention, and relearning phases were analysed using a repeated-measures mixed-effects model with a Geisser–Greenhouse correction (fixed effects: condition, time, condition × time; random effect: mouse) to account for missing sessions for some mice and violation of sphericity (see Section 4.6). EBC transition phases were analysed using either a repeated-measures mixed-effects model (fixed effects: condition, time, condition × time; random effect: mouse) for Fc and NRP1 mice to account for missing sessions, or a repeated-measures two-way ANOVA (fixed effects: condition, time, condition × time; random effect: mouse) for WT and K108N mice. A Šídák-adjusted multiple comparisons test was used to perform pairwise comparisons on individual EBC days for each parameter. The memory retention-2 phase was analysed using an unpaired two-tailed *t*-test, as it consisted of a single time point. Unpaired two-tailed *t*-tests were used to analyse the EBC Δ %CR. Because the Fc data from Fc and NRP1 mice were skewed, a Mann–Whitney test was used. Iba1 data from Fc and NRP1 mice were analysed using an unpaired two-tailed *t*-test with Welch’s correction due to unequal standard deviation (SD). Gephyrin density and VGLUT1, VGLUT2, and VGAT size and density were analysed using unpaired two-tailed *t*-tests. Pearson’s correlations were calculated to assess the interactions between VGLUT1, VGLUT2 and VGAT size and density. Linear regression analyses were performed for each size–density correlation. Additional Pearson’s correlations were performed between EBC Δ %CR and terminal size, EBC Δ %CR and terminal density, %CR on D15 for CS trials and terminal size, and %CR on D15 for CS trials and terminal density, separately for VGLUT1, VGLUT2, and VGAT. Sema3A^+^ PNN intensity was analysed using an unpaired two-tailed *t*-test. EBC phases and transition phases are reported as mean ± standard error of the mean (SEM). EBC Δ %CR are reported as mean ± SD. Histological data are reported as mean ± SD. Data are considered significantly different at * *p* < 0.05, ** *p* < 0.01, *** *p* < 0.001 and **** *p* < 0.0001. Statistical power was calculated using G-power software (version 3.1.9.7).

## Data Availability

Data not contained within this article or the Supplementary Materials are available upon request.

## Supplementary Materials

The following supporting information can be downloaded at: https://www.mdpi.com/article/10.3390/ijms27125304/s1.
