# Supplementary material for: Impact of Sema3A Interference on Cerebellum-Dependent Motor Associative Learning and Memory
Source: Int J Mol Sci. 2026 Jun 11;27(12):5304. doi: 10.3390/ijms27125304 (PMC13299268; doi:10.3390/ijms27125304)
Supplement: Supplementary file 1 [file ijms-27-05304-s001.zip › ijms-4309704-supplementary.pdf]

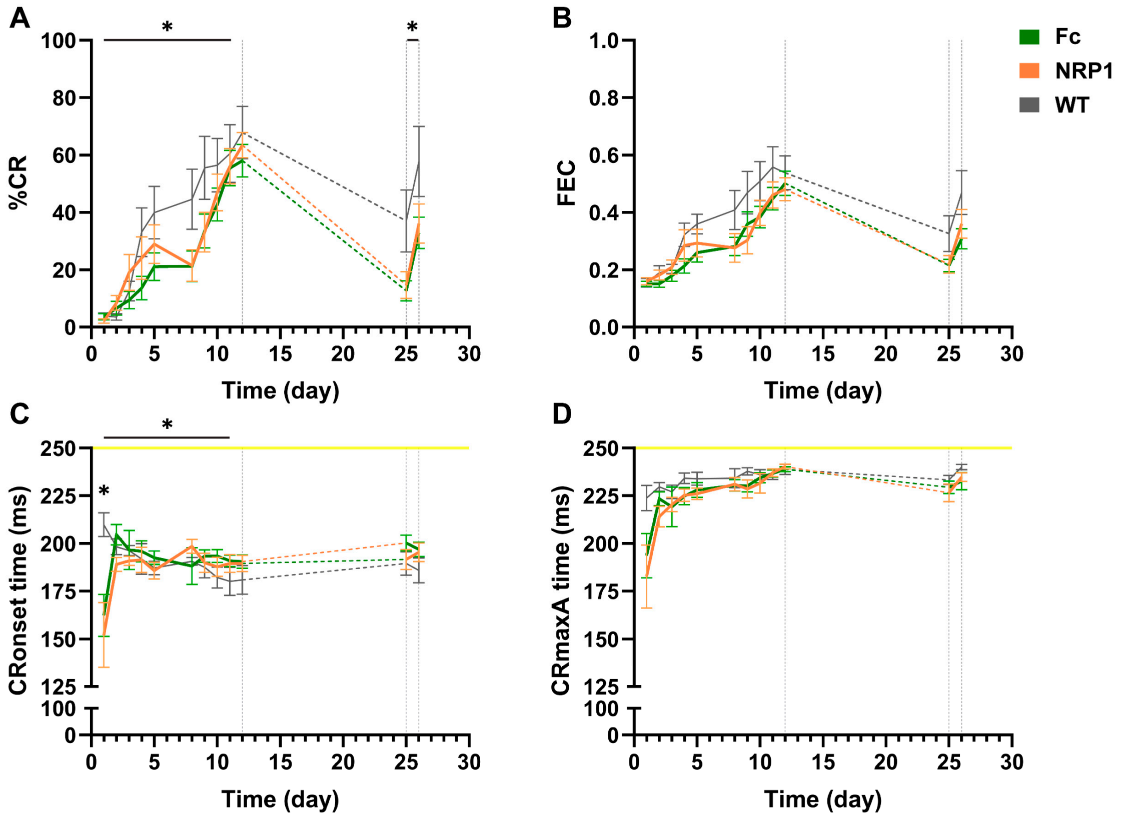

Supplementary Figure S1. EBC performance for paired trials of Fc, NRP1 and WT. (A-D) EBC performance of Fc, NRP1 and WT mice, shown as (A) %CR, (B) FEC, (C) CRonset time and (D) CRmaxA time for CS trials. Dashed lines separate learning (D1-12), memory retention (D15-25) and relearning (D25-26) phases (learning phase: Fc *N mice* = 17, NRP1 *N mice* = 17, WT *N mice* = 7; relearning phase: Fc *N mice* = 17, NRP1 *N mice* = 15, WT *N mice* = 7). Yellow line indicates the US period (C, D). Data is shown as mean ± SEM. * $p < 0.05$.

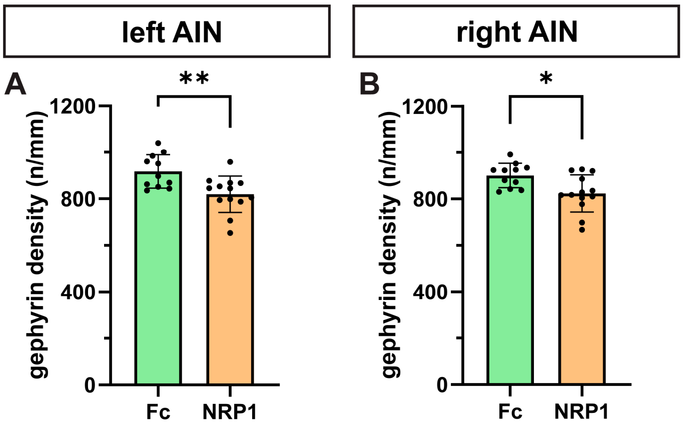

Supplementary Figure S2. Gephyrin density around AIN neurons in the left and right hemisphere of Fc and NRP1 mice. (A, B) Gephyrin density in the ipsilateral (left AIN; A) and contralateral (right AIN; B) sides of the US in Fc and NRP1 mice (per side: Fc *N mice* = 11, NRP1 *N mice* = 13). Data is shown as mean ± SD. * $p < 0.05$, ** $p < 0.01$.

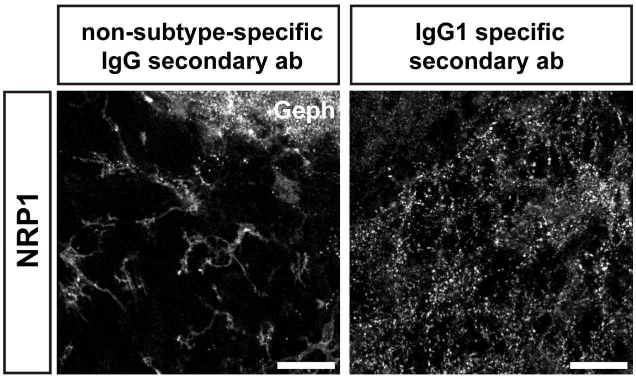

Supplementary Figure S3. Microglia are non-specifically stained in NRP1 mice when using a non-subtype-specific anti-mouse IgG secondary antibody in combination with a mouse anti-gephyrin primary antibody. (A-B) Gephyrin staining in NRP1 mice with an IgG (A) and an IgG1 subtype-specific (B) secondary antibody against the mouse anti-gephyrin primary antibody (ab). Scale bar: A, B: 25µm.

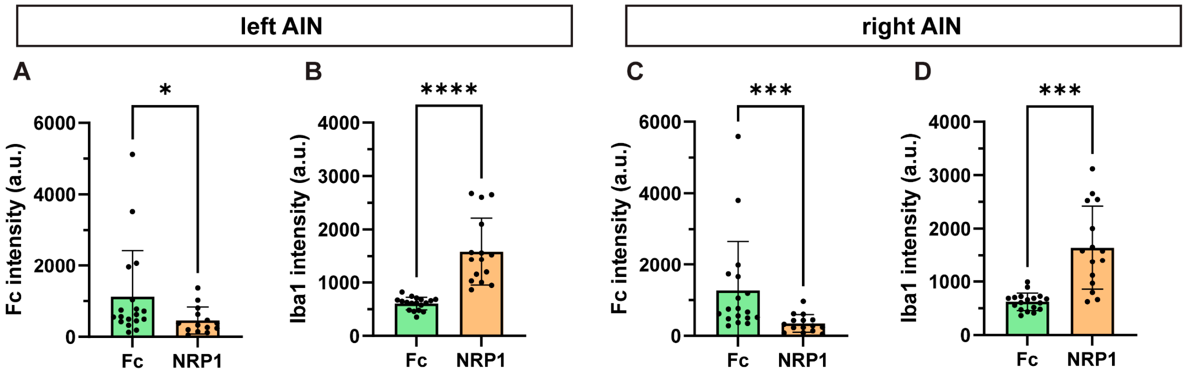

Supplementary Figure S4. Fc and Iba1 intensities in the left and right AIN of Fc and NRP1 mice. (A-D) Fc intensity in the ipsilateral (left AIN; A) and contralateral (right AIN; C) sides of the US in Fc and NRP1 mice (left side, Fc *N mice* = 18, NRP1 *N mice* = 14; right side, Fc *N mice* = 18, NRP1 *N mice* = 15). Iba1 intensity in the ipsilateral (left; B) and contralateral (right; D) sides of the US in Fc and NRP1 mice (left side, Fc *N mice* = 18, NRP1 *N mice* = 15; right side Fc *N mice* = 18, NRP1 *N mice* = 15). Data is shown as mean ± SD. * $p < 0.05$, *** $p < 0.001$, **** $p < 0.0001$.

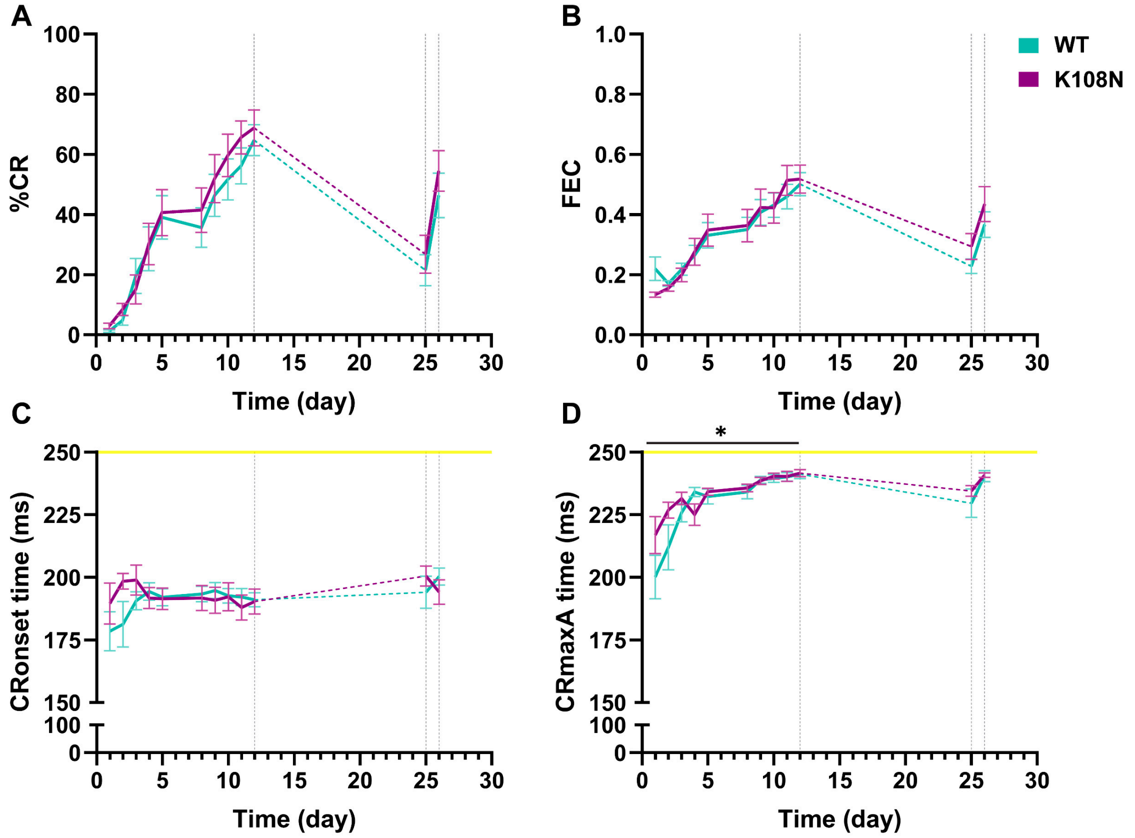

Supplementary Figure S5. EBC performance for paired trials of WT and K108N mice. (A-D) EBC performance of WT and K108N mice, shown as (A) %CR, (B) FEC, (C) CRonset time and (D) CRmaxA time for CS trials. Dashed lines separate learning (D1-12), memory retention (D15-25), relearning (D25-26) and memory retention-2 (D29) phases (learning phase: WT *N mice* = 18, K108N *N mice* = 17; relearning phase: WT *N mice* = 18, K108N *N mice* = 15). Yellow line indicates the US period (C, D). Data is shown as mean ± SEM. * $p < 0.05$.

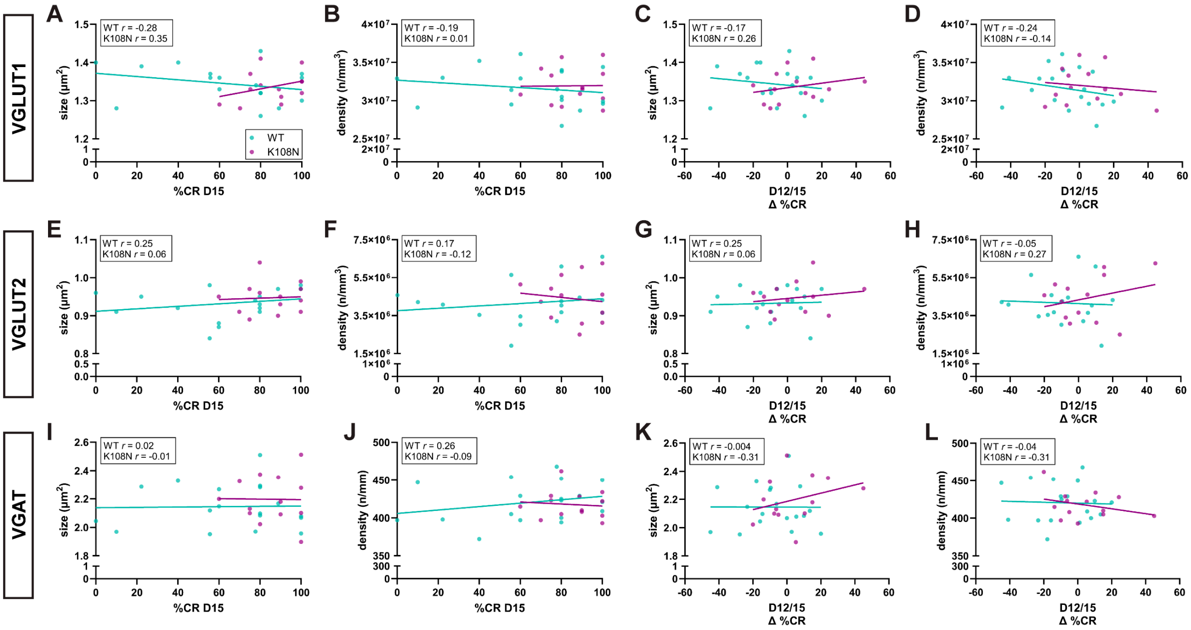

Supplementary Figure S6. Correlations between synapse size or density and the %CR on D15 and Δ %CR during the transition from the learning phase to the memory retention phase for WT and K108N mice. Analysis was focused on the AIN ipsilateral to the US. **(A-D)** Correlation matrix between size or density of VGLUT1$^+$ terminals and the %CR on D15 (A, B) or the Δ %CR during the D12 to D15 transition (C, D) in WT and K108N mice (WT *N mice* = 18, K108N *N mice* = 13). **(E-H)** Correlation matrix between size or density of VGLUT2$^+$ terminals and the %CR on D15 (E, F) or the Δ %CR during the D12 to D15 transition (G, H) in WT and K108N mice (WT *N mice* = 17, K108N *N mice* = 13) **(I-L)** Correlation matrix between size or density of VGAT$^+$ terminals and the %CR on D15 (**I**, J) or the Δ %CR during the D12 to D15 transition (K, L) in WT and K108N mice (WT *N mice* = 18, K108N *N mice* = 13).
